# Bakteriophagen zur Behandlung muskuloskelettaler Infektionen – Eine Übersicht zu klinischem Einsatz, offenen Fragen und rechtlichem Rahmen

**DOI:** 10.1007/s00132-025-04690-z

**Published:** 2025-07-31

**Authors:** Markus Rupp, Tristan Ferry, Mohammadali Khan Mirzaei, Volker Alt, Li Deng, Nike Walter

**Affiliations:** 1 Klinik und Poliklinik für Unfall‑, Hand- und Wiederherstellungschirurgie, Universitätsklinikum Gießen, Rudolf-Buchheim-Straße 7, 35385 Gießen, Deutschland; 2 Institut für Virologie, Helmholtz Zentrum München – Deutsches Forschungszentrum für Gesundheit und Umwelt, Neuherberg, Deutschland; 3 Referenzzentrum für die Behandlung komplexer Knochen- und Gelenkinfektionen (CRIOAc Lyon), Hospices Civils de Lyon, Lyon, Frankreich; 4 Abteilung für Infektionskrankheiten, Hospices Civils de Lyon, Lyon, Frankreich; 5 TUM School of Life Sciences, Lehrstuhl für Prävention mikrobieller Erkrankungen, Zentralinstitut für Infektionsprävention (ZIP), Technische Universität München, Freising, Deutschland; 6 Klinik und Poliklinik für Unfallchirurgie, Universitätsklinikum Regensburg, Regensburg, Deutschland; 7 Justus-Liebig-Universität Gießen, Gießen, Deutschland

**Keywords:** Antibiotika, Endoprothesen, Multiresistenz, Phagentherapie, Protheseninfektionen, Antibiotics, Endoprosthesis, Multidrug resistance, Phage therapy, Periprosthetic joint infection

## Abstract

**Hintergrund:**

Bakteriophagen, kurz Phagen, sind hochspezifische Viren, die gezielt Bakterien infizieren und lysieren. Ihre Wiederentdeckung als therapeutische Option gewinnt angesichts zunehmender Antibiotikaresistenzen an Dynamik. In der Orthopädie und Unfallchirurgie, einem Fachgebiet mit hoher Prävalenz chronischer und implantatassoziierter Infektionen, rückt die Phagentherapie zunehmend in den Fokus. Die gezielte Anwendung – lokal, systemisch oder kombiniert – eröffnet neue Möglichkeiten insbesondere bei komplexen, multiresistenten oder chirurgisch schwer zugänglichen Infektionen.

**Aktuelle Entwicklungen:**

Die historische Entwicklung der Phagentherapie reicht bis ins frühe 20. Jahrhundert zurück, verlor jedoch im Zuge der Antibiotikaentwicklung an Bedeutung. Heute zeigen moderne Studien und Einzelfallberichte vielversprechende Ergebnisse – etwa bei periprothetischen Infektionen – und belegen das Potenzial individualisierter, genetisch charakterisierter Phagencocktails. Auch neue Applikationsformen wie hydrogelbasierte Trägersysteme oder minimal-invasive intraartikuläre Injektionen finden zunehmend Anwendung.

**Rechtliche Situation:**

Regulatorisch bestehen jedoch erhebliche Hürden: Phagen gelten in der EU als biologische Arzneimittel, was komplexe Zulassungsverfahren erfordert. Neben der magistralen Herstellung erlaubt der individuelle Heilversuch den therapeutischen Einsatz bei fehlenden Alternativen. Für eine standardisierte klinische Anwendung bedarf es jedoch evidenzbasierter Protokolle, strukturierter Phagenbanken und enger interdisziplinärer Zusammenarbeit.

**Fazit:**

Die Etablierung der Phagentherapie als komplementäres Instrument in der orthopädischen Infektionsbehandlung erfordert nicht nur regulatorische Klarheit, sondern auch gezielte Forschung, klinische Studien und verantwortungsvolle Anwendung in spezialisierten Zentren.

## Abstract (en)

**Background:**

Bacteriophages, or simply phages, are viruses that specifically infect and lyse bacteria. As antibiotic resistance becomes a growing global concern, phage therapy is gaining renewed attention as a targeted and effective treatment option. In orthopedics and trauma surgery—disciplines characterized by high rates of chronic and implant-associated infections—phages offer promising new therapeutic avenues, particularly in cases involving multidrug-resistant pathogens or when conventional surgery is limited.

**Current developments:**

Originally developed in the early 20th century, phage therapy lost prominence with the rise of antibiotics. Today, case studies and emerging clinical protocols—especially in the context of periprosthetic joint infections—demonstrate the potential of personalized phage cocktails and advanced delivery systems, such as hydrogels or intra-articular injections. Their specificity enables bacterial eradication while sparing the host microbiome.

**Regulatory situation:**

However, the regulatory landscape in the European Union poses significant challenges. Phages are classified as biological medicinal products, requiring comprehensive preclinical and clinical evaluation. While magistral production and compassionate use permit individual applications, broad clinical integration demands standardized protocols, accessible phage libraries, and robust quality control. Furthermore, safety concerns, including immunogenicity and systemic distribution, must be addressed through structured research.

**Conclusion:**

To fully realize the potential of phage therapy in orthopaedic infection management, interdisciplinary collaboration, targeted funding, and clinical trials are essential. Responsible implementation within expert centers can ensure efficacy and safety, paving the way for a controlled and evidence-based adoption of phages as a complementary strategy alongside traditional surgical and antibiotic treatments.

Bakteriophagen rücken als gezielte Waffe gegen multiresistente Infektionen wieder in den Fokus. Der Beitrag beleuchtet historische Entwicklungen, moderne Applikationsformen und regulatorische Rahmenbedingungen mit besonderem Blick auf die Orthopädie und Unfallchirurgie. Dabei wird deutlich: Die Anwendung sollte kontrolliert und wissenschaftlich begleitet erfolgen, um das therapeutische Potenzial nicht durch undifferenzierten Einsatz zu gefährden.

## Bakteriophagen – alte Hoffnung neu entdeckt

**Abb. 1:**
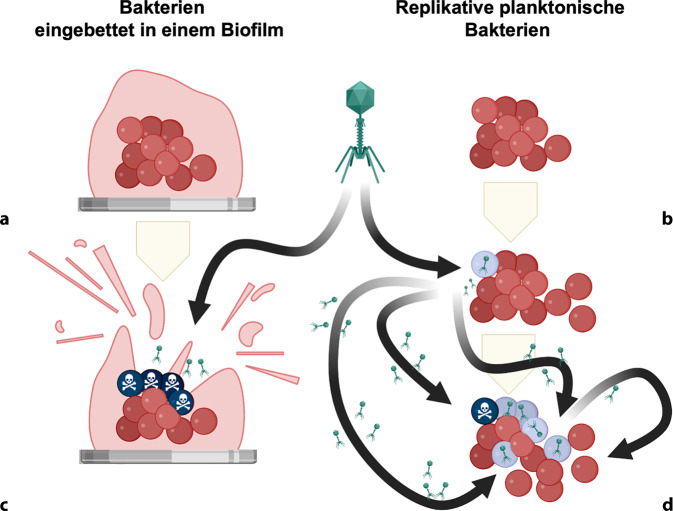
Es existieren zwei unterschiedliche bakterielle Phänotypen pathogener Erreger, die für muskuloskelettale Infektionen verantwortlich sind (**a**, **b**), sowie entsprechende Aktivitäten von Phagen gegen beide Formen (**c**, **d**). Während der Entstehung einer Infektion bilden pathogene Bakterien häufig Biofilme, insbesondere bei Kontakt mit Implantaten, wobei die Biofilme im Verlauf der Zeit weiter ausreifen (**a**). Bakteriophagen zeigen hierbei eine dosisabhängige antibiofilmische Wirkung, die sowohl die Struktur des Biofilms als auch die darin enthaltene bakterielle Biomasse beeinflusst (**c**). Parallel dazu existieren die Erreger auch als replizierende, planktonische Bakterien (**b**). Aktive Phagen sind in der Lage, diese replizierenden Bakterien (dargestellt in *Blau*) zu infizieren, deren zelluläre Mechanismen zu übernehmen und große Mengen neuer Viruspartikel zu produzieren. Durch die Lyse der Wirtszellen (dargestellt in *Schwarz*) können diese neuen Phagen wiederum benachbarte replizierende Bakterien infizieren. Dieser Vorgang ist hochdynamisch und wird geprägt durch die Konkurrenz zwischen der Vermehrung der pathogenen Bakterien und der Ausbreitung der Phagen innerhalb der gesamten bakteriellen Population (**d**). Modifiziert nach [15], © Springer Nature

Bakteriophagen, kurz Phagen, sind Viren, die gezielt Bakterien infizieren und abtöten. Sie gelten als die am häufigsten vorkommenden biologischen Einheiten der Erde [9, 13]. Aufgrund ihrer hohen Spezifität gegenüber bestimmten Bakterienstämmen und ihrer Fähigkeit, resistente Erreger zu eliminieren, rücken sie wieder verstärkt in den Fokus der medizinischen Forschung. Phagen bestehen aus einer Proteinhülle, die das genetische Material umschließt, und weisen vielfältige Formen und Größen auf [37, 45]. In der Therapie werden sie gezielt eingesetzt, um bakterielle Infektionen zu bekämpfen – insbesondere dort, wo Antibiotika versagen ([12, 29]; Abb. 1).

Die Bindung eines Phagen an ein Bakterium erfolgt über spezifische Rezeptoren auf der bakteriellen Zelloberfläche [21]. Phagen, die ihren Wirt letztlich töten – auch virulente Phagen genannt –, schleusen ihr Erbmaterial in die Wirtszelle ein, vermehren sich dort und führen zur Zerstörung der Bakterienzelle – neue Phagen werden freigesetzt und setzen den Infektionszyklus fort [47]. Durch diese gezielte Wirkweise greifen Phagen nur spezifische Erreger an und schonen dabei die natürliche Mikroflora. Vor allem bei multiresistenten oder chronischen Infektionen, bei denen klassische Antibiotika versagen, bieten sie einen vielversprechenden Behandlungsansatz [37, 38].

## Historische Ursprünge der Bakteriophagentherapie

Die Idee, Phagen zur Behandlung bakterieller Infektionen einzusetzen, reicht bis ins späte 19. Jahrhundert zurück. Bereits 1896 berichtete der britische Bakteriologe Ernest Hanbury Hankin über antimikrobielle Aktivitäten in Flusswasser in Indien [1]. Der Begriff „Bakteriophage“ wurde 1917 von dem französischen Mikrobiologen Félix d’Hérelle geprägt, der auch als einer der Begründer der Phagentherapie gilt. Zwei Jahre später gelang ihm eine erfolgreiche Behandlung von vier Kindern mit bakterieller Ruhr am Hôpital des Enfants-Malades in Paris – eines der frühesten dokumentierten Anwendungsbeispiele [39].

Im Jahr 1923 gründete George Eliava, ein Schüler von Félix d’Hérelle am Institut Pasteur in Paris, gemeinsam mit seinem Lehrer in seinem Heimatland Georgien das Eliava-Institut in Tiflis (Tbilissi), das sich zu einem international anerkannten Zentrum für Phagenforschung entwickelte [8]. Auch in der Sowjetunion und in Polen, insbesondere am Hirszfeld-Institut in Breslau, wurde die Therapie weiterverfolgt. Zwischen 1981 und 1999 wurden dort rund 1900 Patienten behandelt, mit Erfolgsquoten von über 80 % [35, 43].

## Rückgang und Renaissance der Phagentherapie

Mit dem Aufstieg der Antibiotika nach dem Zweiten Weltkrieg verlor die Phagentherapie in der westlichen Welt an Bedeutung. Die Entdeckung des Penicillins durch Alexander Fleming und die rasante Verbreitung von Antibiotika in Medizin, Landwirtschaft und Tierhaltung läuteten eine neue Therapieära ein [2]. Gleichzeitig entstanden zunehmend Probleme durch multiresistente Erreger [11].

Heute gelten antibiotikaresistente Infektionen als eine der größten globalen Herausforderungen. Jährlich sterben weltweit bereits über 1,2 Mio. Menschen an den Folgen resistenter Infektionen, bis 2050 könnte diese Zahl auf 10 Mio. ansteigen [26]. Die Phagentherapie erlebt in diesem Kontext eine Wiederentdeckung. Ihre hohe Spezifität, die Möglichkeit zur Individualisierung und das Potenzial, auch bei resistenten Keimen wirksam zu sein, machen sie zu einer vielversprechenden Ergänzung zur Antibiotikatherapie.

## Frühere Anwendungsformen bei orthopädischen Infektionen

Vor der breiten Verfügbarkeit von Antibiotika wurde die lokale Phagenapplikation insbesondere bei trauma- oder kriegsbedingten Infektionen genutzt. Der französische Orthopäde André Raiga-Clémenceau, ein Schüler von d’Hérelle, wies jedoch auf die begrenzte Wirksamkeit der Phagenbehandlung bei Knochennekrosen hin. Seiner Ansicht nach könnten Phagen zwar die Ausbreitung der Infektion aufhalten, jedoch kein abgestorbenes, nicht mehr durchblutetes Knochengewebe retten, das operativ entfernt werden müsse [17].

Mit der Weiterentwicklung von operativen Verfahren zur Knochenheilung und Gelenkrekonstruktion verlagerte sich die Behandlung bakterieller Infektionen in der Orthopädie und Unfallchirurgie zunehmend auf chirurgische Maßnahmen. Ziel ist neben der mechanischen Sanierung die Entfernung des Biofilms und die Identifikation der Erreger, gefolgt von einer gezielten Antibiotikatherapie [34].

In Osteuropa, vor allem in Georgien, Polen und Russland, blieb die Phagentherapie jedoch erhalten. Sie wurde meist ambulant und ohne chirurgische Begleitmaßnahmen angewandt, oft mit nicht pharmazeutisch hergestellten Phagen [23, 25]. Bei Patienten mit septischer Pseudarthrose, kortikaler oder endomedullärer Osteomyelitis kamen kombinierte Verfahren aus lokaler Phagenapplikation auf die Wunde sowie oraler Einnahme über mehrere Wochen zum Einsatz. Ziel war es, durch die lokale Replikation der Phagen tiefere Infektionsherde zu erreichen und über den Blutweg zusätzliche Wirkung zu erzielen. Aufgrund fehlender chirurgischer Sanierung und unklarer systemischer Verfügbarkeit wurden diese Ansätze jedoch nicht als optimale Therapie angesehen.

## Moderne Applikationswege in der Orthopädischen Chirurgie

**Abb. 2:**
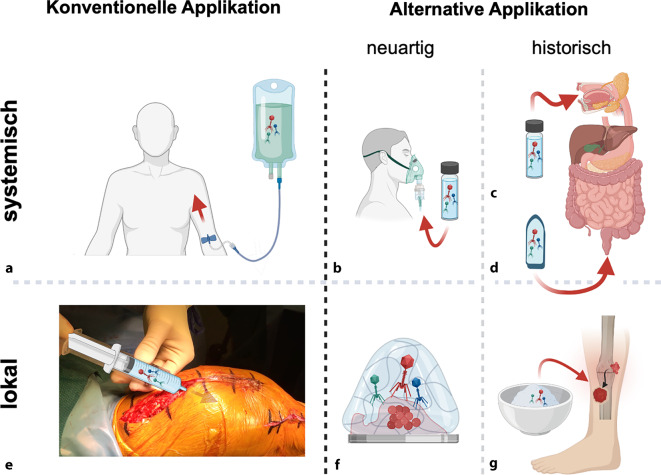
Verschiedene Verabreichungswege, die bei Patienten mit muskuloskelettalen Infektionen aktuell Anwendung finden oder künftig genutzt werden könnten. Systemische Verabreichung: **a** Die intravenöse Applikation stellt die klassische Methode der systemischen Verabreichung dar. **b** Die Verneblung ist ein neuer und vielversprechender Ansatz der systemischen Applikation, der weiter erforscht werden sollte. Die Verabreichung über den Gastrointestinaltrakt, entweder oral (**c**) oder rektal (**d**), wird historisch als mögliche Form der systemischen Verabreichung betrachtet. Dabei könnten Phagen über den Darm in den Blutkreislauf gelangen und so das infizierte Knochengewebe erreichen. Lokale Verabreichung: **e** Die direkte Anwendung von Phagen in flüssiger Form direkt an der Infektionsstelle gilt als etablierter Standard der lokalen Therapie. **f** Der Einsatz innovativer Formulierungen wie Hydrogelen, die eine einfachere Applikation während eines chirurgischen Eingriffs an einem infizierten Implantat ermöglichen und eine verlängerte lokale Freisetzung der Phagen begünstigen könnten, ist von großem Interesse (**g**) und kann insbesondere für die lokale Verabreichung eine wichtige Rolle spielen. Diese Ansätze müssen weiterentwickelt, klinisch evaluiert und als alternative Verabreichungsform betrachtet werden. Modifiziert nach [15], © Springer Nature

Heute gelten zwei Applikationswege als konventionell: die systemische intravenöse Gabe von pharmazeutisch hergestellten Phagen sowie die lokale Applikation intraoperativ oder durch gezielte Injektionen ([16, 17]; Abb. 2). Für die intravenöse Anwendung sind Phagen in pharmazeutischer Qualität Pflicht, um Sicherheitsrisiken zu minimieren. Weitere mögliche systemische Wege umfassen die Inhalation mittels Vernebler sowie die orale oder rektale Gabe [7, 25, 31].

Die lokale Anwendung im Rahmen eines chirurgischen Eingriffs ist in der Theorie besonders geeignet, birgt aber praktische Herausforderungen aufgrund der anatomischen Vielfalt. Zusätzlich wird die topische Anwendung bei infizierten Wunden als alternative Methode diskutiert [28]. Eine interessante Entwicklung ist die Kombination mit hydrogelen Trägersystemen, die intraoperativ auf Implantatoberflächen appliziert werden und dort über einen definierten Zeitraum Phagen freisetzen [3, 5].

## Phagentherapie bei Protheseninfektionen

Infektionen von Gelenkprothesen („periprosthetic joint infection“ [PJI]) gelten als besonders vielversprechendes Anwendungsfeld der Phagentherapie. In akuten Fällen (< 3 Monate nach Inokulation) wird aktuell ein implantaterhaltendes Vorgehen favorisiert, da sich der Biofilm zu diesem Zeitpunkt noch nicht stabilisiert hat [22, 49]. Hier kommt das sogenannte DAIR-Verfahren (Debridement, Antibiotika, Implantaterhalt) zum Einsatz: Debridement, Austausch der Polyethylen-Komponente, ausgiebige Lavage sowie antibiotische Therapie [27, 32].

Bei chronischen Infektionen ist meist ein ein- oder zweizeitiger Prothesenwechsel angezeigt, was jedoch mit hoher Morbidität und Funktionsverlust verbunden sein kann [24]. Daher wird die Phagentherapie insbesondere für Patienten diskutiert, bei denen eine Explantation vermieden werden soll oder eine komplizierte Wechseloperation nicht möglich erscheint.

Ein bekanntes Beispiel ist das „Haverty-Protocol“ der Mayo Clinic (USA), bei dem eine chronische Knieinfektion mit *Klebsiella pneumoniae* allein durch 40 intravenöse Phagenapplikationen erfolgreich behandelt wurde [6]. Daraufhin etablierte die Klinik ein eigenes Programm zur Phagentherapie bei PJI (Clinical Trials Identifier: NCT05314426).

Weitere klinische Protokolle wurden z. B. in Baltimore umgesetzt: entweder als Kombination aus einer lokalen Applikation mit 5‑tägiger intravenöser Therapie oder als 5‑tägige kombinierte lokale und systemische Behandlung. Dabei wurde auch auf potenzielle Komplikationen durch Kathetereinsatz hingewiesen, insbesondere bei Knieprothesen [14].

**Abb. 3:**
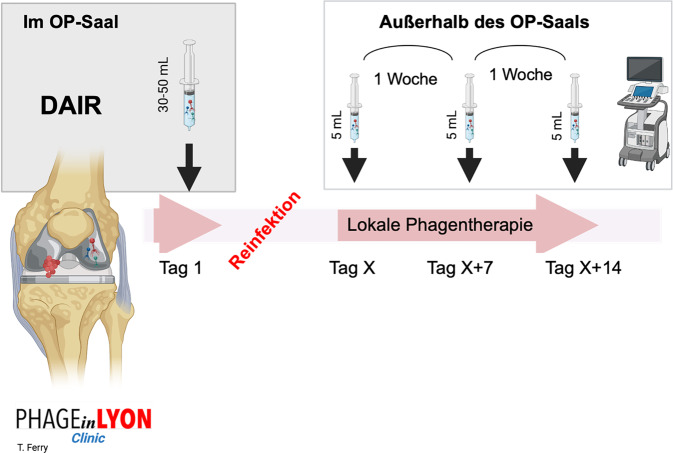
Behandlung von Patienten mit einer Protheseninfektion, die nach dem initialen „PhagoDAIR“-Verfahren ein Rezidiv erlitten haben und anschließend im Rahmen des PHAGEinLYON-Programms ausschließlich aktive Phageninjektionen unter sonographischer Kontrolle erhielten (drei Injektionen im Abstand von jeweils einer Woche). Das Vorgehen illustriert probate Möglichkeiten der lokalen intra- und postoperativen Applikation; *DAIR* Debridement, Antibiotika, Implantaterhalt. Modifiziert nach [15], © Springer Nature

Ein innovatives Verfahren stellt das „PhagoDAIR“-Konzept dar, das in Lyon entwickelt wurde. Hier wird bei chronischen oder rezidivierenden Infektionen ein klassisches DAIR durchgeführt, wobei am Ende des Eingriffs eine intraartikuläre Einzelgabe eines Phagencocktails erfolgt. Anschließend werden während der suppressiven Antibiotikatherapie drei sonographiegesteuerte Injektionen im Wochenabstand durchgeführt ([17, 18]; Abb. 3). Diese Methode ist minimal-invasiv, katheterfrei und erlaubt die gleichzeitige Entnahme von Gelenkflüssigkeit zur Kontrolle. Sie wurde in die Studie „PhagoDAIR I“ (NCT05369104) als Rettungsmaßnahme integriert.

In Fällen, in denen eine Operation nicht möglich ist, wurde auch eine rein konservative Strategie erprobt, bestehend aus täglicher intravenöser und alle 2 Tage lokaler Phagengabe über mehrere Wochen. Erste Ergebnisse zu Sicherheit und Langzeitwirkung dieser Ansätze werden derzeit ausgewertet.

## Spezielle Aspekte der Anwendung und offene Fragen

Trotz der wachsenden Bedeutung der Bakteriophagentherapie bei der Behandlung komplexer Infektionen in der Orthopädie und Unfallchirurgie bleibt das konventionelle Vorgehen – bestehend aus konsequenter chirurgischer Sanierung in Kombination mit einer systemischen antibiotischen Therapie – unverzichtbarer Behandlungsstandard. Die Phagentherapie ist als additive Maßnahme zu verstehen, die das bestehende Therapiekonzept sinnvoll ergänzen kann. Insbesondere der wiederholt nachgewiesene Synergieeffekt zwischen Antibiotika und Phagen sollte gezielt genutzt werden [20, 33]. Hierzu empfiehlt sich eine vor Therapiebeginn durchgeführte In-vitro-Testung auf additive oder synergistische Wirkungen. Um der Ausbildung phagenresistenter Bakterienstämme entgegenzuwirken, sollte – wann immer möglich – auf standardisierte oder patientenspezifisch zusammengestellte Phagencocktails zurückgegriffen werden [47].

Trotz vielversprechender Fallberichte sind zentrale klinische Parameter bislang unzureichend definiert. Insbesondere bestehen offene Fragen hinsichtlich der optimalen Applikationsform (lokal und/oder systemisch, intraoperativ vs. postoperativ), der Frequenz und Dauer der Anwendung sowie der erforderlichen Konzentration und des Applikationsvolumens. Die Etablierung evidenzbasierter Standards setzt prospektive klinische Studien voraus, welche diese Variablen systematisch untersuchen.

Ein weiterer zentraler Aspekt ist die Therapiesicherheit: Obwohl schwerwiegende Nebenwirkungen bislang selten dokumentiert wurden, fehlt es an umfassenden Daten zu potenziellen immunologischen Reaktionen, systemischer Phagenverteilung oder Interaktionen mit dem menschlichen Organismus. Zur Sicherstellung der Patientensicherheit sollten im Rahmen klinischer Anwendungen gezielte Sicherheitsuntersuchungen erfolgen, wie etwa der Nachweis von Phagen im Blut nach lokaler Applikation oder immunologische Tests zur Erfassung möglicher Antikörperbildung und zellulärer Reaktionen. Dies sind unter anderem die Hauptgründe, weshalb aus gebotenem Patientenschutz regulatorische Hürden bei der Anwendung von Bakteriophagen einzuhalten sind.

## Regulatorische Situation in der EU und Deutschland

### Zulassungshürden für Phagentherapeutika

In der Europäischen Union gelten therapeutisch eingesetzte Bakteriophagen als biologische Arzneimittel im Sinne der Richtlinie 2001/83/EG [20, 33]. Für ihre Anwendung ist grundsätzlich eine behördliche Zulassung erforderlich. Diese Vorgabe wurde in Deutschland durch das Arzneimittelgesetz (AMG) umgesetzt. Die Einstufung von Bakteriophagen als biologische Arzneimittel macht eine vollständige behördliche Zulassung erforderlich, bei der strenge Anforderungen an Qualität, Sicherheit und Wirksamkeit erfüllt werden müssen. Neben der aufwendigen präklinischen und klinischen Prüfung stellt insbesondere die Herstellung nach GMP(„good manufacturing practice“)-Standards eine große Hürde dar, da sie hohe technische, personelle und finanzielle Ressourcen erfordert, die bisher nur sehr wenige Einrichtungen bereitstellen können. Zudem ist die Bewertung individualisierter Phagenpräparate im standardisierten Zulassungsverfahren schwierig, da ihre Zusammensetzung häufig patienten- und infektspezifisch angepasst werden muss, was mit dem klassischen Zulassungsrahmen kaum vereinbar ist.

### Magistrale Herstellung von Bakteriophagen

Eine Ausnahme von der Zulassungspflicht besteht gemäß § 21 Absatz 2b AMG für Arzneimittel, die in einer Apotheke auf ärztliche Verschreibung hin für einen bestimmten Patienten individuell hergestellt werden – im Rahmen der sogenannten magistralen Zubereitung. Diese Sonderregelung erlaubt es, Phagen ohne formale Zulassung therapeutisch einzusetzen, solange die Herstellung patientenbezogen erfolgt.

Trotz Zulassungsfreiheit müssen diese individuell hergestellten Arzneimittel laut § 55 Absatz 8 AMG den anerkannten pharmazeutischen Regeln entsprechen. Dazu zählen unter anderem die Vorgaben der Europäischen Pharmakopöe (Ph. Eur.), dem offiziellen Arzneibuch der EU sowie die Einhaltung der GMP. Diese Standards sind entscheidend, um Qualität, Sicherheit und Wirksamkeit auch bei nichtindustriellen Herstellungsvorgängen zu gewährleisten. Dazu zählen unter anderem die Identifizierung des Phagenstammes, meist durch Sequenziermethoden, die Wirksamkeitsprüfung meist durch „Plaque-assay“-Testung und die Prüfung auf Reinheit. Bei letzterem werden Verunreinigungen wie Endotoxine, die eine Immunreaktion auslösen können, untersucht.

Derzeit wird in der Ph. Eur. ein eigenes Kapitel zur Qualitätskontrolle von Phagentherapeutika erarbeitet. Ziel ist es, europaweit einheitliche und verbindliche Anforderungen für Phagenarzneimittel zu etablieren.

### Anwendung im Rahmen von „compassionate use“/individuellem Heilversuch

Neben der magistralen Zubereitung besteht die Möglichkeit, Bakteriophagen auch außerhalb einer regulären Zulassung im Rahmen eines sogenannten „compassionate use“ anzuwenden. Dabei ist zwischen strukturierten „Compassionate-use“-Programmen und dem individuellen Heilversuch im Einzelfall zu unterscheiden. Während erstere gemäß Artikel 83 der Verordnung (EG) Nr. 726/2004 zentral koordiniert und in Deutschland durch § 21 Absatz 2 Nr. 6 AMG geregelt sind – und in der Regel eine Genehmigung durch das Bundesinstitut für Arzneimittel und Medizinprodukte (BfArM) erfordern –, handelt es sich beim individuellen Heilversuch um eine therapeutische Maßnahme außerhalb der Zulassung, die ohne formale Anzeige oder Genehmigung durchgeführt werden kann.

Der individuelle Heilversuch erlaubt es behandelnden Ärzten, ein nicht zugelassenes Arzneimittel wie ein Phagenpräparat auf eigene Verantwortung anzuwenden, wenn alle zugelassenen Therapien ausgeschöpft oder nicht verfügbar sind. Diese Vorgehensweise beruht auf dem Prinzip der therapeutischen Freiheit und setzt das Einverständnis des Patienten voraus. Sie entspricht der Definition eines „unerfüllten medizinischen Bedarfs“ nach Artikel 37 der Deklaration von Helsinki der Weltärztevereinigung (WMA), die unbewiesene Interventionen in der klinischen Praxis erlauben, sofern ein begründeter wissenschaftlicher Nutzen erwartet werden kann. Wann genau alle therapeutischen Optionen ausgeschöpft sind, ist bei Knochen- und Gelenkinfektionen nicht genau definiert. In der Praxis wird die Notwendigkeit der Bakteriophagentherapie meist mit dem Vorhandensein von multiresistenten Erregern oder einem drohenden Extremitätenverlust begründet. Hier müssen in Zukunft noch genauere Richtlinien etabliert werden.

Auch im Rahmen des individuellen Heilversuchs gelten Anforderungen an die Qualität und Sicherheit des verwendeten Phagenpräparats. Eine Herstellung nach GMP ist zwar nicht zwingend vorgeschrieben, aber aus fachlicher Sicht wünschenswert, insbesondere im Hinblick auf Identität, Reinheit (z. B. Endotoxingehalte) und mikrobiologische Unbedenklichkeit. Die Verantwortung für Indikation, Auswahl und Anwendung liegt dabei vollständig beim behandelnden Arzt.

Diese Einschränkungen verdeutlichen die Notwendigkeit von regulatorischen Modellen, die besser mit den biologischen und klinischen Besonderheiten der Phagentherapie übereinstimmen. Die derzeitigen Rahmenbedingungen, die auf festen Formulierungen und standardisierten Studiendesigns basieren, sind zumindest für die personalisierte Natur der Phagentherapie ungeeignet. In diesem Zusammenhang stellen adaptive Zulassungsmodelle eine vielversprechende Alternative dar. Diese Modelle ermöglichen eine gestufte Bewertung von Sicherheit und Wirksamkeit, die Einbindung von Real-World-Daten sowie iterative Anpassungen der therapeutischen Zusammensetzung. Eine solche Flexibilität ist für die Phagentherapie entscheidend, da Behandlungen häufig individuell auf bestimmte Krankheitserreger abgestimmt und an Resistenzentwicklungen angepasst werden müssen. Regulatorische Strategien, die diese biologischen und klinischen Komplexitäten berücksichtigen, könnten eine breitere Anwendung der Phagentherapie ermöglichen, ohne die Patientensicherheit oder die behördliche Aufsicht zu gefährden [10].

In Deutschland kann sich der behandelnde Arzt bei entsprechender Indikation aktuell an spezialisierte Zentren mit Erfahrung in der Phagentherapie wenden. Zusätzlich kann in Zusammenarbeit mit einer Apotheke eine magistrale Herstellung nach ärztlicher Verschreibung erfolgen. Auch ist ein individueller Heilversuch nach § 21 Abs. 2b AMG möglich. Eine genaue Dokumentation, die informierte Einwilligung des Patienten und eine interdisziplinäre Abstimmung (z. B. mit Infektiologie, Mikrobiologie, Klinikapotheke) sind essenziell. Die Beratung durch Institutionen wie das BfArM oder Fachgesellschaften kann zusätzlich unterstützen.

Die Kosten für eine Phagentherapie im Rahmen individueller Heilversuche sind derzeit nicht regelhaft erstattungsfähig und müssen in der Regel vom behandelnden Zentrum oder Patienten selbst getragen werden. Eine Erstattung durch die Krankenkasse ist nur in Einzelfällen im Rahmen einer Kostenübernahmevereinbarung möglich.

## Zukunftsperspektiven und Forschung

Die Phagentherapie gewinnt zunehmend an Bedeutung als ergänzende oder alternative Option zur Behandlung bakterieller Infektionen – insbesondere im Kontext zunehmender Antibiotikaresistenzen. Besonderes Potenzial bietet die Orthopädie und Unfallchirurgie, da in unserem Fachgebiet eine hohe Zahl an chronischen und implantatassoziierten Infektionen, etwa im Rahmen periprothetischer Infektionen, frakturassoziierter Infektionen und Osteomyelitiden, auftritt [30, 34, 40, 42]. Diese Infektionen stellen eine erhebliche klinische Herausforderung dar und sind häufig langwierig, komplikationsträchtig und mit eingeschränkten Therapieoptionen verbunden [4, 36, 41].

Die lokal begrenzte, aber gleichzeitig präzise applizierbare Phagentherapie ist in unserem Fachgebiet besonders gut einsetzbar, etwa in Form von lokal aufgebrachten Phagenlösungen oder Trägermaterialien während operativer Revisionen [3]. Durch diese hohe klinische Relevanz und gute Anwendbarkeit kommt der Orthopädie und Unfallchirurgie eine wichtige Rolle in der Weiterentwicklung und Etablierung der Phagentherapie zu – sowohl im Rahmen von klinischer Forschung als auch in der Anwendung individueller Heilversuche. Eine enge interdisziplinäre Abstimmung ist daher notwendig, um den klinischen Einsatz sicher und wirksam zu gestalten, und die Etablierung der Phagentherapie vom experimentellen Therapieansatz bis hin zur Standardbehandlung schwierig zu behandelnder muskuloskelettaler Infektionen zu erreichen und weiterzuentwickeln [46]. Ein zentraler Baustein für die zukünftige Etablierung einer Phagentherapie ist neben der Bewältigung regulatorischer Hürden der Aufbau strukturierter Phagenbanken, die eine schnelle Verfügbarkeit geprüfter und charakterisierter Phagenstämme ermöglichen. Parallel dazu wird an der Entwicklung standardisierter Phagencocktails geforscht, die gegen häufige Erreger gerichtet sind, breiter eingesetzt werden und die Phagenresistenzgefahr verringern können [47].

Langfristig bieten insbesondere individualisierte Therapieansätze großes Potenzial, bei denen die Auswahl der Phagen auf Basis genetischer Erregeranalysen erfolgt. Moderne molekularbiologische Verfahren ermöglichen es, gezielt gegen bakterielle Resistenzen vorzugehen und maßgeschneiderte Behandlungsstrategien zu entwickeln [44]. Darüber hinaus eröffnet die Kombination mit biotechnologischen Methoden, etwa durch gentechnisch veränderte oder CRISPR-modifizierte Phagen, neue therapeutische Möglichkeiten, zum Beispiel zur gezielten Abschaltung bakterieller Resistenzgene [19].

## Fazit für die Praxis

- Die Phagentherapie ist in der Orthopädie und Unfallchirurgie eine vielversprechende Ergänzung zur konventionellen Infektionsbehandlung, vor allem bei komplexen, multiresistenten oder chronischen Verläufen.
- Damit dieses Potenzial nicht durch unkontrollierte oder undifferenzierte Anwendung gefährdet wird, sollte der Einsatz auf spezialisierte Zentren konzentriert werden. Nur dort kann die Therapie unter standardisierten Bedingungen durchgeführt, dokumentiert und evaluiert werden, um langfristig belastbare Daten zur Wirksamkeit und Sicherheit zu generieren.
- Ein unkritischer Einsatz ohne strukturierte Begleitung birgt die Gefahr, die Glaubwürdigkeit dieser Therapieform zu untergraben und wertvolle therapeutische Chancen in Zukunft ungenutzt zu lassen.
- Eine kontrollierte, evidenzbasierte Integration der Phagentherapie in die Infektionsbehandlung erfordert interdisziplinäre Zusammenarbeit, regulatorische Klarheit und gezielte Forschungsförderung.

## Abkürzungsverzeichnis

AMG: Arzneimittelgesetz
CRISPR: Clustered Regularly Interspaced Short Palindromic Repeats
DAIR: „Debridment, antibiotics, implant retention“
EU: Europäische Union
GMP: gute Herstellungspraxis („good manufacturing practice“)
Ph. Eur.: Europäische Pharmakopöe (europäisches Arzneibuch)
PJI: Periprothetische Gelenkinfektion (periprosthetic joint infection)
WMA: Weltärztebund (World Medical Association)
